# Main complications and results of treatment with intra-arterial infusion chemotherapy through the subclavian and thoracic arteries for locally advanced breast cancer

**DOI:** 10.3892/mco.2013.129

**Published:** 2013-05-23

**Authors:** XIAOYI WANG, CHANGING GAN, HONGYUAN LI, YUXIAN WEI, DONCHANG ZHU, GUANGLUN YANG, XINLIANG SU, JEAN-FRANÇOIS RODIER, GUOSHENG REN

**Affiliations:** 1 Department of Breast and Endocrine Surgery, First Affiliated Hospital, Chongqing Medical University, Chongqing 400016, P.R. China;; 2 Department of Surgical Oncology, Paul Strauss Cancer Center, 67065 Strasbourg, France

**Keywords:** locally advanced breast cancer, efficacy and complication, intra-arterial chemotherapy, subclavian artery, thoracic artery

## Abstract

Intra-arterial infusion chemotherapy for locally advanced breast cancer (LABC) has been previously performed. However, the main complications of this type of chemotherapy remain to be clarified. In the present study, catheterization chemotherapy was carried out for 53 LABC cases (stage IIIa–IIIc) between May, 2006 and March, 2007. For IIIB and IIIC patients, the catheters were guided to the opening of the subclavian artery. For stage IIIa patients, the catheters were placed into the thoracic artery through a subcutaneous femoral artery puncture. One to four cycles of chemotherapy (mean, 1.6 cycles) were administered for the patients using taxotere, epidoxorubicin, 5-fluorouracil and/or cyclophosphamide. The interval time between the two cycles was 21 days. Seven cases were identified as complete response (CR, 13.2%), 41 cases were partial response (PR, 77.4%) with a rate of effectiveness of (CR + PR, 90.6%), 5 cases were stable disease (SD, 9.40%) and no case was progressive. Pain of the ipsilateral upper extremity was present in 7 cases. Two cases exhibited ipsilateral upper extremity atrophy following drug administration from the opening of the subclavian artery. One case experienced neck pain and headache, while in one case necrosis of local skin was evident. Hematological toxicity over grade 3 was observed in 6 cases (11.30%). Systemic toxicity was mild and did not affect the quality of life of the patients. Overall survival was identified as 18/51 (35.3%), and free-disease survival as 10/51 (19.6%). In conclusion, intra-arterial infusion chemotherapy is an effective local control treatment for LABC. The main complications are pain of the ipsilateral upper extremity and neck as well as headache. Severe complications are ipsilateral upper extremity atrophy and necrosis of local skin. During the treatment, controlling the pressure of the tourniquet and velocity of drug administration are crucial for reducing local complications.

## Introduction

Locally advanced breast cancer (LABC) is defined as patients who present with the clinical stages of T4 N0-1M0 and T3N1 (stage IIIa), T0-3 N2M0 (stage IIIb), and T0-4 N3M0 (stage IIIc), without distant metastasis. T and N staging were as per the 6th edition of the American Joint Committee on Cancer, the TNM staging classification and revision of the American Joint Committee on Cancer staging system for breast cancer (1). LABC is characterized by large breast tumors involving the skin or muscles of the chest wall and extensive involvement of the local lymph nodes. Therefore treatment involves combined modality including systemic chemotherapy, surgery and radiotherapy (2,3).

Good local control and down-staging increase the possibility for surgery. Intra-arterial infusion is suggested to take advantage of the first pass effect of chemotherapeutics, generating higher local drug concentrations at the tumor cell membrane and therefore enhancing cellular drug uptake. Drug exposure to the tumor starts at the time of drug uptake through the cell membrane. Studies have focused on intra-arterial infusion chemotherapy for LABC and results have demonstrated high local control (4–9). However, the main complications of intra-arterial infusion chemotherapy have yet to be reported. In the present study, we report on the results and main complications of intra-arterial infusion chemotherapy through the subclavian and thoracic arteries for 53 LABC cases for the period December, 2006 to December, 2010. The results demonstrated the efficacy of good local control for LABC and revealed serious complications with intra-arterial infusion chemotherapy.

## Materials and methods

### Subjects

The patients with LABC were recruited from the Department of Breast and Endocrine Surgery, The First Affiliated Hospital of Chongqing Medical University, between December, 2006 and December, 2010. Clinical stage IIIa was identified in 10 patients, stage IIIb in 24 patients and stage IIIc in 19 patients. The patients were female with a mean age of 47.7 years (range, 28–67 years). Histological examination confirmed carcinoma of the breast (left side in 30 and right side in 23) by pathological diagnosis via fine needle biopsy pre-operative intra-arterial infusion chemotherapy. The findings of the chest X-ray, ventral ultrasonography, radioisotope scan and head nuclear magnetic resonance did not provide evidence of distant metastasis. This study was approved by the Ethics Committee of Chongqing Medical University. All patients provided written consent.

### Treatment

**Figure 1.**
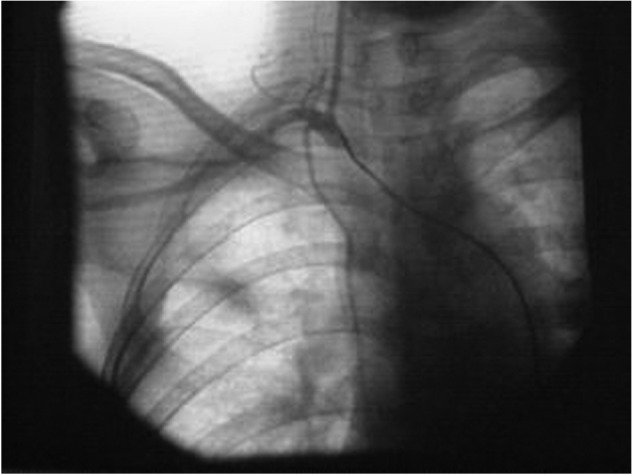
For patients in stage IIIb and IIIc, the catheter was guided to the opening of the subclavian and lateral thoracic arteries. Prior to drug administration, a contrast agent (iohexol) was injected to comfirm the location of the catheter. An arteriogram of the subclavian, thoracic and vertebral arteries was obtained.

**Figure 2.**
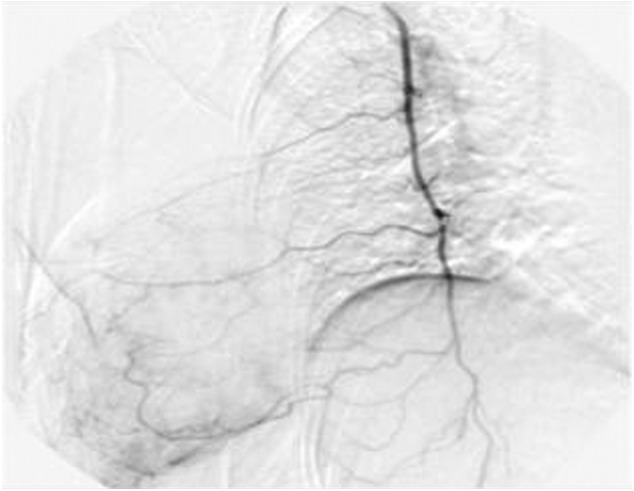
Arteriogram of the thoracic artery. For patients in stage IIIa, the catheter was inserted into the thoracic artery. Prior to drug administration, contrast agent (iohexol) was injected to comfirm the location of the catheter.

The patients were treated with angiography (Siemens Coroskop Plus, Germany) under local anesthesia. According to the Seldinger technique, a 6F catheter was inserted percutaneously into the femoral artery. Subsequently, the catheter was guided to the opening of the subclavian artery. An arteriogram of the subclavian, thoracic and vertebral arteries was obtained for the patients (Fig. 1). For patients in stage IIIa, the catheter was inserted into the thoracic artery (Fig. 2). For patients in stage IIIb and IIIc, the catheter was guided to the opening of the subclavian and lateral thoracic arteries (Fig. 1). Prior to drug administration, contrast agent (iohexol) was injected to comfirm the location of the catheter again and the ipsilateral upper arm was bundled with a tourniquet, at a pressure of 260–280 mmHg.

Drug doses administered included taxotere (100 mg/m^2^), epidoxorubicin (100 mg/m^2^), 5-fluorouracil (1,000 mg) and/or cyclophosphamide (800 mg/m^2^). The tumor response, local lymph nodes and occurrence of local or systemic complications determined the number of cycles of chemotherapy for which there was an interval time of 21 days between two cycles.

### Response criteria

Based on the WHO criteria the response was estimated according to the clinical features following treatment and all the patients were evaluated by computerised tomography scan preoperation. Complete disappearance of all the lesions was considered a complete response (CR); macroscopic reduction in size by ≥50% was considered a partial response (PR); a reduction of 25–50% was designated as stable disease (SD); and the appearance of any new lesions not previously identified or an estimated increase of 25% in existent lesions was considered progressive disease.

## Results

Of the 53 patients, 7 cases (13.2%) were CR; 41 cases (77.4%) were PR, with a rate of effectiveness of (CR + PR: 90.6%, 48/53); 5 cases (9.4%) were SD and no case was progressive. The treatment results of LABC are provided in Table I.

### Main complications

Complications occurred mainly in the local areas. Pain of the ipsilateral upper extremity was noted in 7 cases. One case experienced neck pain and headache; this patient recovered 2 weeks later without any special treatment. Two cases had ipsilateral upper extremity atrophy and disability and did not recover within a time period of 6 months, following drug administration from the opening of the subclavian and lateral thoracic arteries. One case had necrosis of local skin, but recovered with conservative treatment. The systemic toxicity was mild and did not affect the quality of life of patients (Table I).

### Other complications

Of the 53 patients, no complications related to the angiographic technique were observed. Hematological toxicity over grade 3 such as fever and without bleeding was observed in 6 cases (11.3%), while 9 patients had gastrointestinal symptoms including nausea, vomiting, diarrhea and stomachache. Cardiovascular toxicity was not observed (Table I).

## Discussion

At present, a combination of systemic therapy with locoregional treatment (surgery and/or radiotherapy) constitutes the standard of care in LABC patients since improving locoregional control is associated with improved survival (10). In patients with stage III breast cancer treated with induction chemotherapy followed by surgery, radiotherapy or combination therapy, the risk of locoregional recurrence is at a range of 20% (9). The use of induction systemic therapy results in tumor downstaging and in selected LABC patients even allows for breast conserving surgery (11–14).

Intra-arterial infusion chemotherapy is an effective and safe treatment for the local tumor control of LABC (7). In our data, which also demonstrated the good local control of LABC with intra-arterial infusion chemotherapy, the CR + PR was 90.6%, which was higher than that reported by Shimamoto *et al* (8), who noted that the local response rate was 77.3% (at least more than two regimens) and Pacetti *et al* (7) who noted that the response rate was 80%. Factors such as the drugs used and administration of chemotheraputic cycles likely affected the results of those authors. However, in the present study, treatment involved different methods of drug administration. For patients in stage IIIa, large breast tumors and the skin or muscles of the chest wall were usually involved, thus local control tumors were primary tumors. We inserted a catheter into the thoracic artery and administered the majority of the drugs into the chest area, as there would be more effective local control and downstaging. For patients in stage IIIb and IIIc, the local lymph nodes were usually extensively involved. Control of the regional lymph nodes was considered crucial, therefore, the catheter was guided to the opening of the subclavian and lateral thoracic arteries, allowing more drugs to be administered in the subclavian, superclavian and axilla regions These methods contributed to improving the response rate of local lesions and increased the possibility of surgery. However, results of the follow-up revealed that overall and disease-free survival had not improved.

Few studies have reported on local complications following intra-arterial infusion chemotherapy for LABC. In the present study, severe complications were observed during treatment. Two patients in stage IIIb had ipsilateral upper extremity atrophy leading to disability, and these patients did not recover within a 6-month time period. Pain of the ipsilateral upper extremity was noted in 7 cases that recovered two weeks later without any special treatment. The reasons for these complications included the loosening of the tourniquet during drug administration, which caused the drugs to flow into the ipsilateral upper extremity, and/or rapid drug administration. One patient experienced neck pain and headache, but recovered without any special treatment. The reason for the symptoms involved drugs flowing into the vertebral artery.

Previous studies have reported toxicity with systemic chemotherapy and hematological toxicity over grade 3 in 4–65% of patients (15,16). Of the 53 patients included in the present study, no complications associated with the angiographic technique were observed. Hematological toxicity over grade 3 such as fever and without bleeding was observed in 6 cases (11.3%). The patients were treated with human granulocyte colony-stimulating factor (human GCSF) and recovered. Nine patients had gastrointestinal symptoms including nausea, vomiting, diarrhea and stomachache. The patients were administered timely symptomatic treatment and recovered. Cardiovascular toxicity was not observed.

In conclusion, intra-arterial infusion chemotherapy is an effective treatment for local tumor control and tumor downstaging of LABC, thereby increasing the possibility for surgery. Low systemic toxicity and good patient compliance are also beneficial. However, severe complications may occur during treatment. Thus, controlling the pressure of the tourniquet and velocity of drug administration are crucial for reducing local complications.

## Figures and Tables

**Table I.** Patient characteristics, treatment, response and complications.

No.	Age (years)	Stage	Location	Cycles	Drugs	Response	Complication
1	32	IIIb	Left	2	EPI	PR	No
2	28	IIIb	Right	1	EPI+CTX+5-FU	SD	Inappetence
3	53	IIIb	Right	1	EPI+CTX+5-FU	PR	No
4	67	IIIb	Left	1	EPI	PR	Pain of upper extremity
5	65	IIIb	Left	3	EPI+CTX+5-FU	CR	Inappetence
6	52	IIIb	Right	1	EPI+5-FU	PR	No
7	51	IIIa	Left	1	EPI+CTX+5-FU	PR	No
8	47	IIIa	Right	1	EPI+CTX+5-FU	PR	No
9	28	IIIa	Left	2	T	PR	Hematological toxicity over grade 3
10	32	IIIb	Left	4	T	CR	No
11	57	IIIa	Right	1	EPI+CTX+5-FU	SD	Pain of upper extremity
12	67	IIIc	Left	4	T	CR	Hematological toxicity over grade 3
13	31	IIIa	Left	1	EPI+CTX+5-FU	PR	No
14	34	IIIc	Right	1	EPI+CTX+5-FU	PR	No
15	51	IIIa	Left	1	T	PR	No
16	66	IIIc	Right	1	T	PR	Pain of upper extremity
17	56	IIIc	Right	2	T	PR	Necrosis of local skin
18	41	IIIa	Left	1	T	PR	No
19	46	IIIc	Right	2	EPI+CTX+5-FU	PR	No
20	39	IIIc	Left	2	T	PR	No
21	41	IIIb	Left	2	EPI+CTX+5-FU	PR	No
22	45	IIIa	Left	1	EPI+CTX+5-FU	PR	Pain of upper extremity
23	50	IIIb	Right	1	T	PR	Pain of upper extremity; nausea
24	50	IIIb	Left	2	EPI+CTX+5-FU	PR	No
25	50	IIIc	Left	1	EPI+CTX+T	PR	No
26	49	IIIb	Left	2	EPI+CTX+T	CR	Pain of upper extremity
27	48	IIIb	Left	2	EPI+CTX+T	PR	Diarrhea
28	43	IIIb	Right	1	EPI+CTX+5-FU	PR	No
29	36	IIIc	Left	1	EPI+CTX+5-FU	PR	Nausea
30	36	IIIb	Right	2	EPI+CTX+T	PR	Hematological toxicity over grade 3
31	54	IIIb	Left	1	EPI+CTX+T	PR	No
32	60	IIIc	Right	2	EPI+CTX+T	CR	Pain of upper extremity
33	66	IIIc	Right	1	EPI+CTX+T	PR	Diarrhea
34	53	IIIc	Left	1	EPI+CTX+T	PR	No
35	42	IIIc	Right	1	EPI+CTX+T	PR	No
36	42	IIIb	Right	1	EPI+CTX+T	PR	Stomachache
37	42	IIIa	Right	2	EPI+CTX+T	PR	No
38	47	IIIb	Left	2	EPI+CTX+5-FU	SD	No
39	45	IIIb	Right	3	EPI+CTX+T	CR	Ipsilateral upper extremity atrophy
40	29	IIIb	Right	2	EPI+CTX+T	PR	Hematological toxicity over grade 3
41	53	IIIc	Left	2	EPI+CTX+T	PR	Nausea
42	29	IIIb	Right	2	EPI+CTX+T	PR	No
43	60	IIIc	Left	1	EPI+CTX+T	PR	No
44	61	IIIc	Left	3	EPI+CTX+T	PR	Hematological toxicity over grade 3
45	60	IIIc	Left	1	EPI+CTX+5-FU	SD	No
46	52	IIIb	Left	2	EPI+CTX+5-FU	PR	Pain of neck and headache
47	47	IIIb	Left	1	EPI+CTX+5-FU	SD	No
48	61	IIIc	Left	2	EPI+CTX+T	PR	No
49	62	IIIc	Right	1	T	PR	No
50	54	IIIb	Right	1	EPI+CTX+T	PR	No
51	42	IIIb	Right	2	EPI+CTX+T	PR	Hematological toxicity over grade 3
52	55	IIIa	Left	2	EPI+CTX+T	CR	Vomiting
53	60	IIIb	Left	1	EPI+CTX+T	PR	Ipsilateral upper extremity atrophy

EPI, epidoxorubicin; CTX, cyclophosphamide; 5-FU, 5-fluorouracil; CR, complete response; PR, partial response; SD, stable disease; T, taxotere.
